# Protection of renal tubules against gentamicin induced nephrotoxicity

**DOI:** 10.12861/jrip.2013.03

**Published:** 2013-03-01

**Authors:** Majid Tavafi

**Affiliations:** ^1^Department of anatomy, Faculty of Medicine, Lorestan University of Medical sciences, Khoram Abad, Iran

**Keywords:** Gentamicin, Nephrotoxicity, Metformin, Garlic, Antioxidants

**Implication for health policy/practice/research/medical education:**

It seems that renal tubular damages in acute renal failure involved in gentamicin nephrotoxicity or ischemia/reperfusion mainly induced by increasing of reactive oxygen species (oxidative stress). According to this attitude, many researchers have been used different antioxidant agents in combat with gentamicin nephrotoxicity. Treatment of animal with metformin against gentamicin revealed that gentamicin might be induced renal tubular damages via energy depletion in renal tubular cells besides inducing of oxidative stress. More studies are needed to clarify renal protective effect of adenosine monophosphate-activated protein kinase (AMPK) activator such as metformin in combat with gentamicin nephrotoxicity.

In spite of undesirable gentamicin nephrotoxicity,‏ this antibiotic is commonly used versus Gram-negative bacteria and still constitutes the only effective therapeutic alternative against microorganisms-pseudomonas, proteus and serratia – that are insensitive to other antibiotics (1). Moreover, gentamicin has been widely used for inducing of acute‏ renal failure in experimental animals and evaluation of renoprotective agents.

The pathological mechanisms involved in gentamicin induced nephrotoxicity include induction of oxidative stress, apoptosis, necrosis, up regulation of transforming growth factor B, elevation of endothelin I , increase of monocyte/macrophages infiltration , phospholipidosis an increase of intracellular sodium ions (2,3). Gentamicin has been showed to increase the generation of super oxide anions, hydroxyl radicals, hydrogen peroxide and reactive nitrogen species in kidney and lead to renal injuries (1).

Gentamicin nephrotoxicity is characterized functionally by an increase of serum creatinine, blood urea nitrogen, and decrease in glomerular filtration rate (4), which morphologically characterized by proximal tubule epithelial desquamation, tubular necrosis, tubular fibrosis, epithelial edema and glomerular hypertrophy (5). Most researchers against gentamicin nephrotoxicity focused on the use of various antioxidants.

More investigations showed that antioxidant agents inhibited or attenuated gentamicin

nephrotoxicity in rats. Usage of antioxidants improved histological injuries such as tubular necrosis, tubular cell edema and apoptosis in gentamicin-injected rats (6-8).

Metformin that used by diabetic patients showed oxidative stress inhibitor activity and adenosine monophosphate-activated protein kinase (AMPK) activator. Some researchers evaluated effects of metformin against gentamicin nephrotoxicity. They reported beneficial effect of metformin in combat with renal histopathological changes induced by gentamicin (8-10).

Alterations in epithelial cell polarity and in the subcellular distributions of epithelial ion transport proteins are key molecular consequences of acute kidney injury and intracellular energy depletion. AMPK, a cellular energy sensor, is rapidly activated in response to renal ischemia, and renal epithelial cells subjected to energy depletion (11).

In the study of Baradaran and colleague for the first time combination effect of metformin and garlic extract evaluated against gentamicin nephrotoxicity. They showed that this treatment attenuated renal histopathological injuries including epithelial cell vacuolization, degeneration, tubular cell flattening, hyaline cast, tubular dilatation, and debris materials in tubular lumen- induced by gentamicin (8). Treatment of animal with metformin against gentamicin revealed that gentamicin might be induced renal tubular damages via energy depletion in renal tubular cells besides inducing of oxidative stress and other mechanisms. More studies are needed to clarify renal protective effect of AMPK activator such as metformin in combat with gentamicin induced nephrotoxicity.

## Acknowledgements

The author wants to thank from the personals of Department of Anatomy, Lorestan University of Medical Sciences.

## Author’s contribution

MT is the single author of the manuscript.

## Conflict of interests

The author declared no competing interests.

## Ethical considerations

Ethical issues (including plagiarism, data fabrication, double publication) have been completely observed by the author.

## Funding/Support

None declared.
